# Supplementary figures and images for: A Comparative Genomics Approach for Analysis of Complete Mitogenomes of Five Actinidiaceae Plants
Source: Genes (Basel). 2022 Oct 9;13(10):1827. doi: 10.3390/genes13101827 (PMC9601400; doi:10.3390/genes13101827)

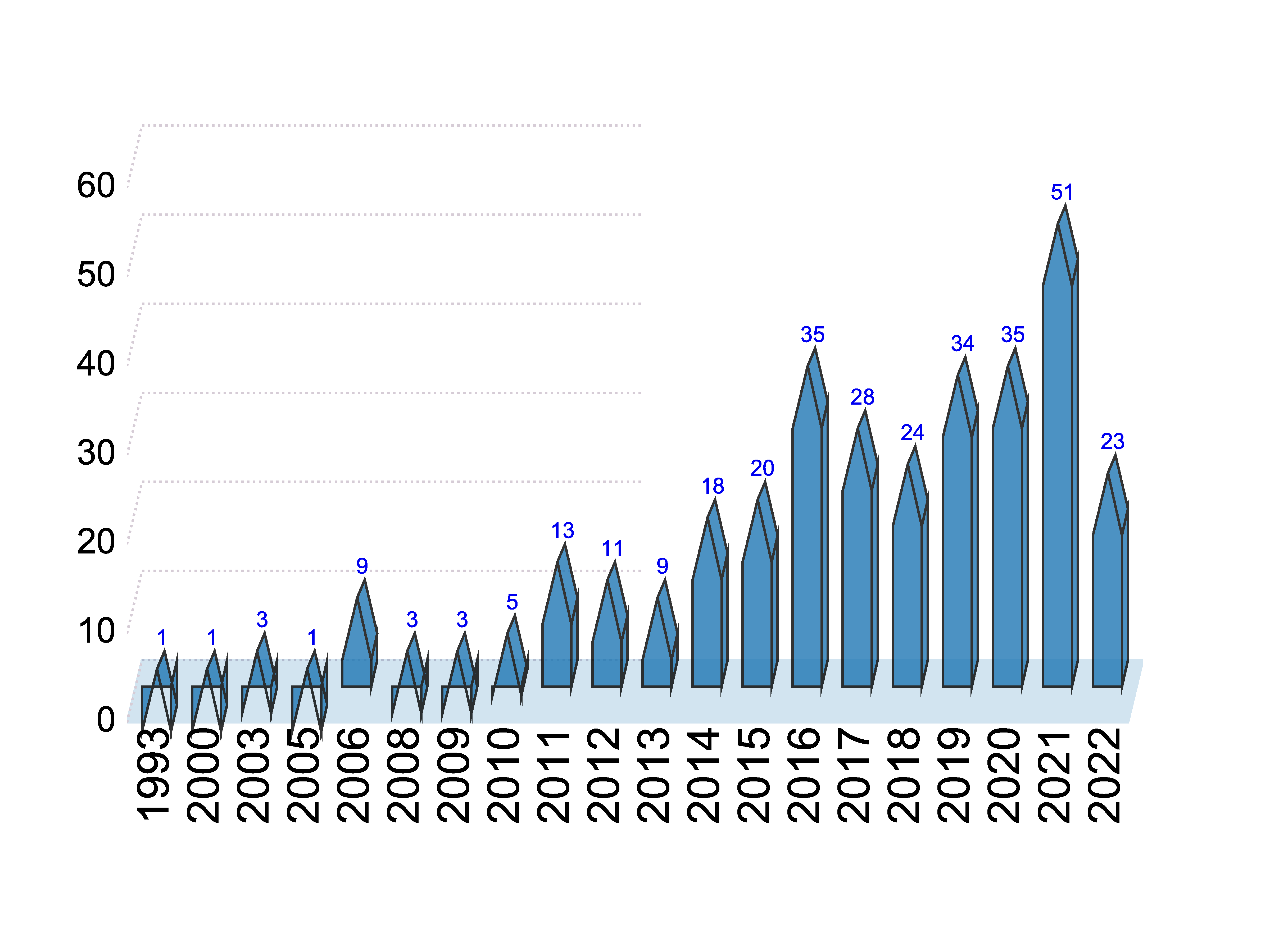

Supplement: Supplementary file 1 [file genes-13-01827-s001.zip › Figure S1 Statistics information of sequenced land plant complete mitogenomes from 1993 to 2022.tiff]

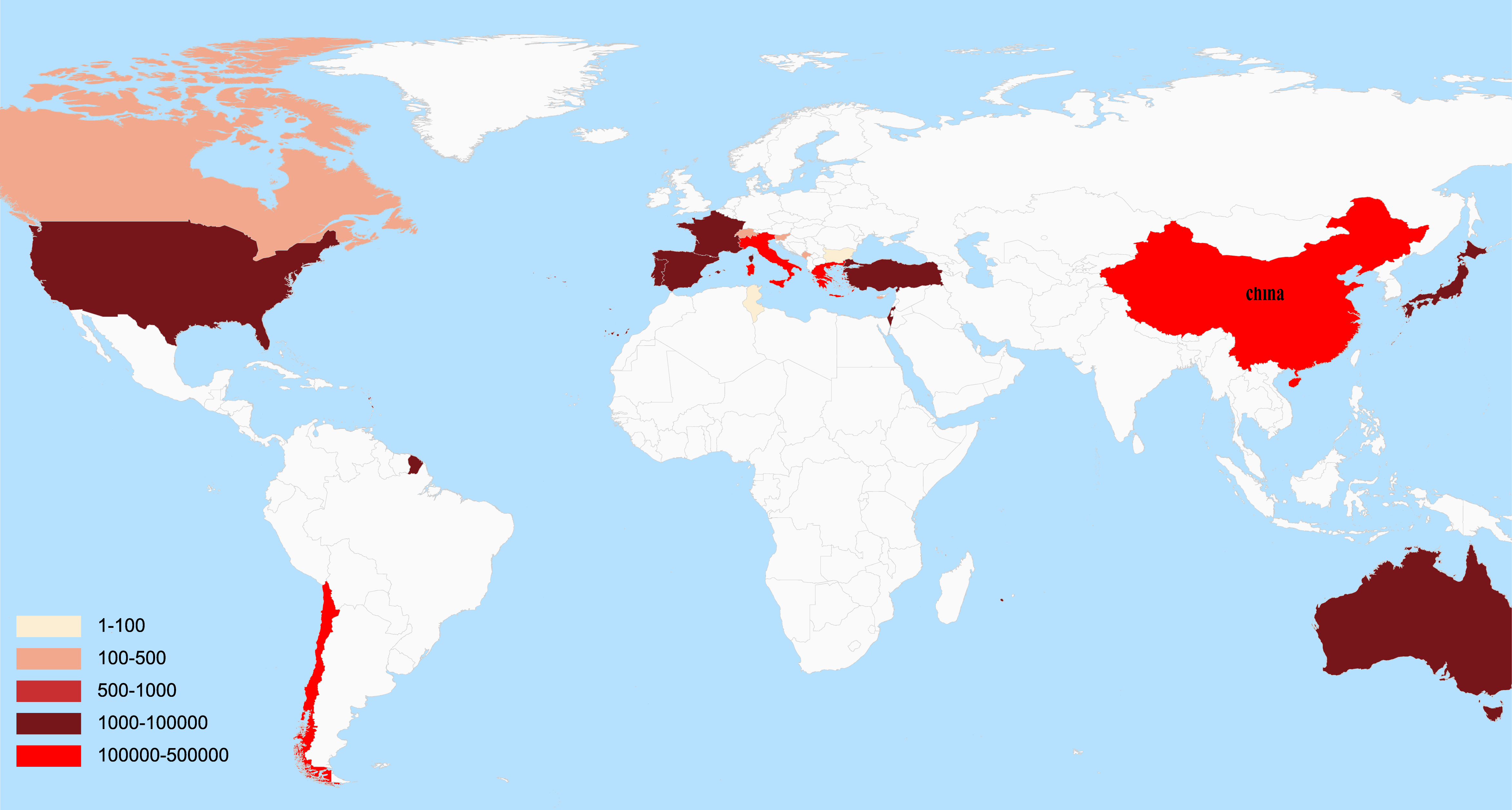

Supplement: Supplementary file 1 [file genes-13-01827-s001.zip › Figure S2 Production quantities of kiwifruit by country.tif]

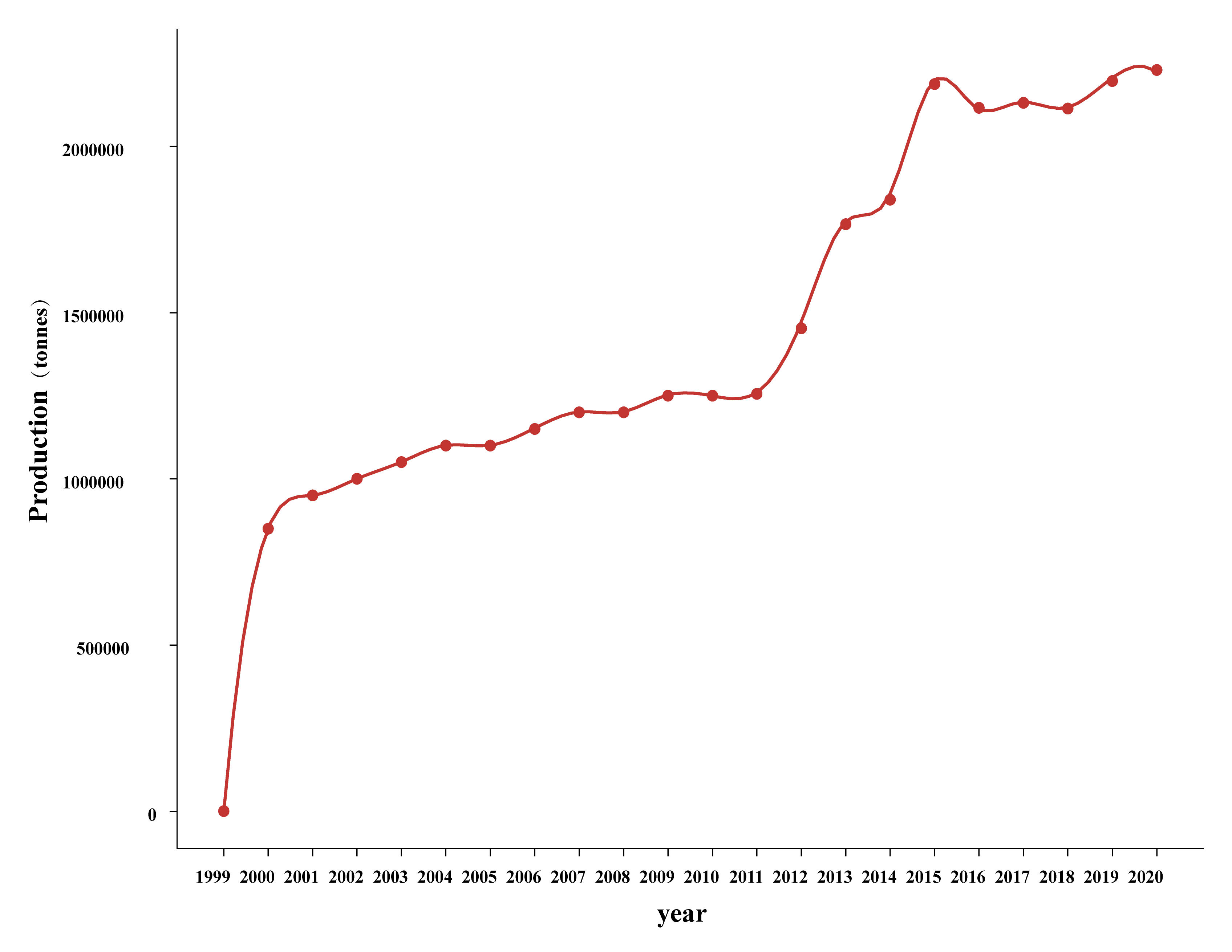

Supplement: Supplementary file 1 [file genes-13-01827-s001.zip › Figure S3 Yield quantities of kiwifruit in world from 1999 to 2020.tif]

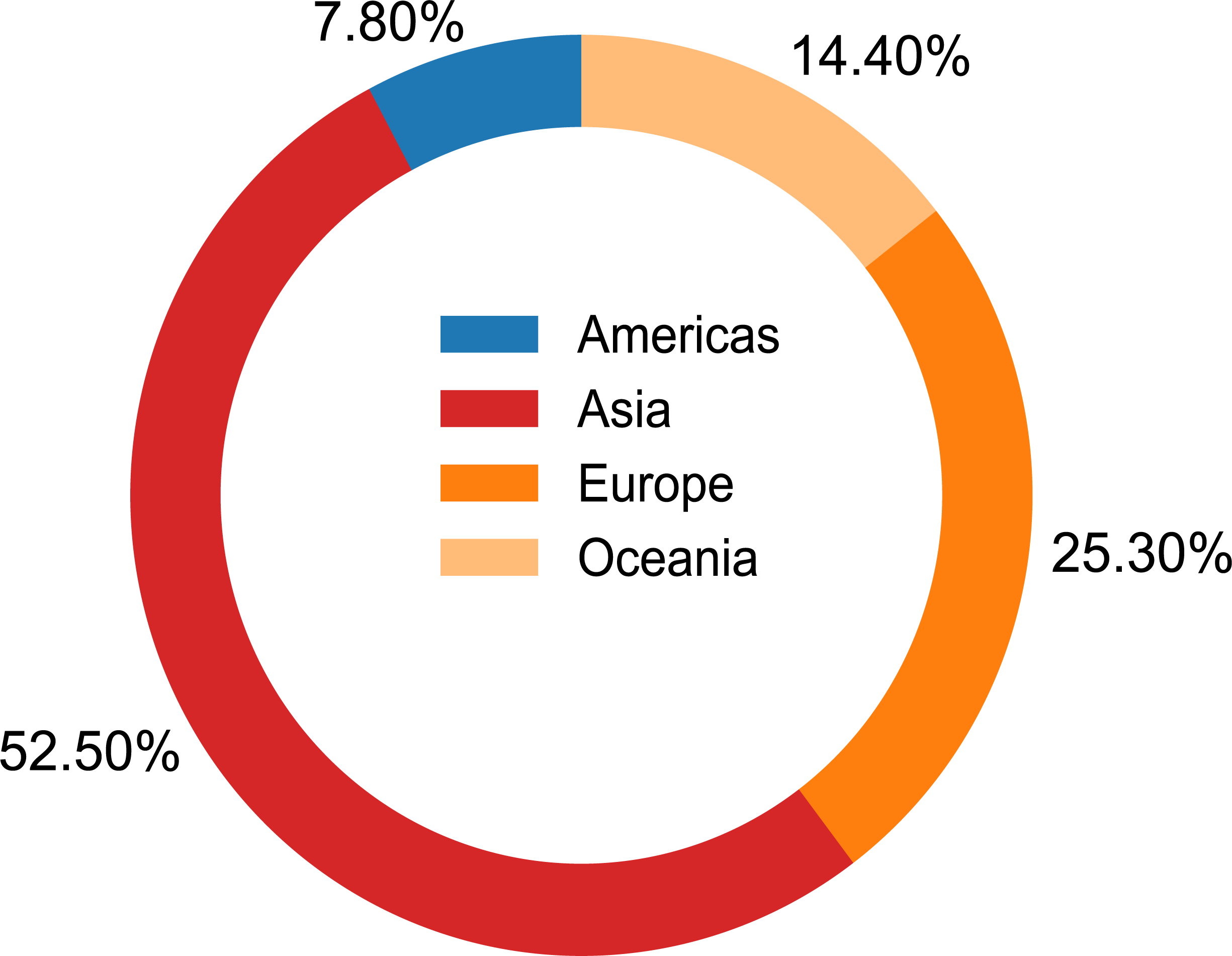

Supplement: Supplementary file 1 [file genes-13-01827-s001.zip › Figure S4 Production share of kiwifruit by region.tif]

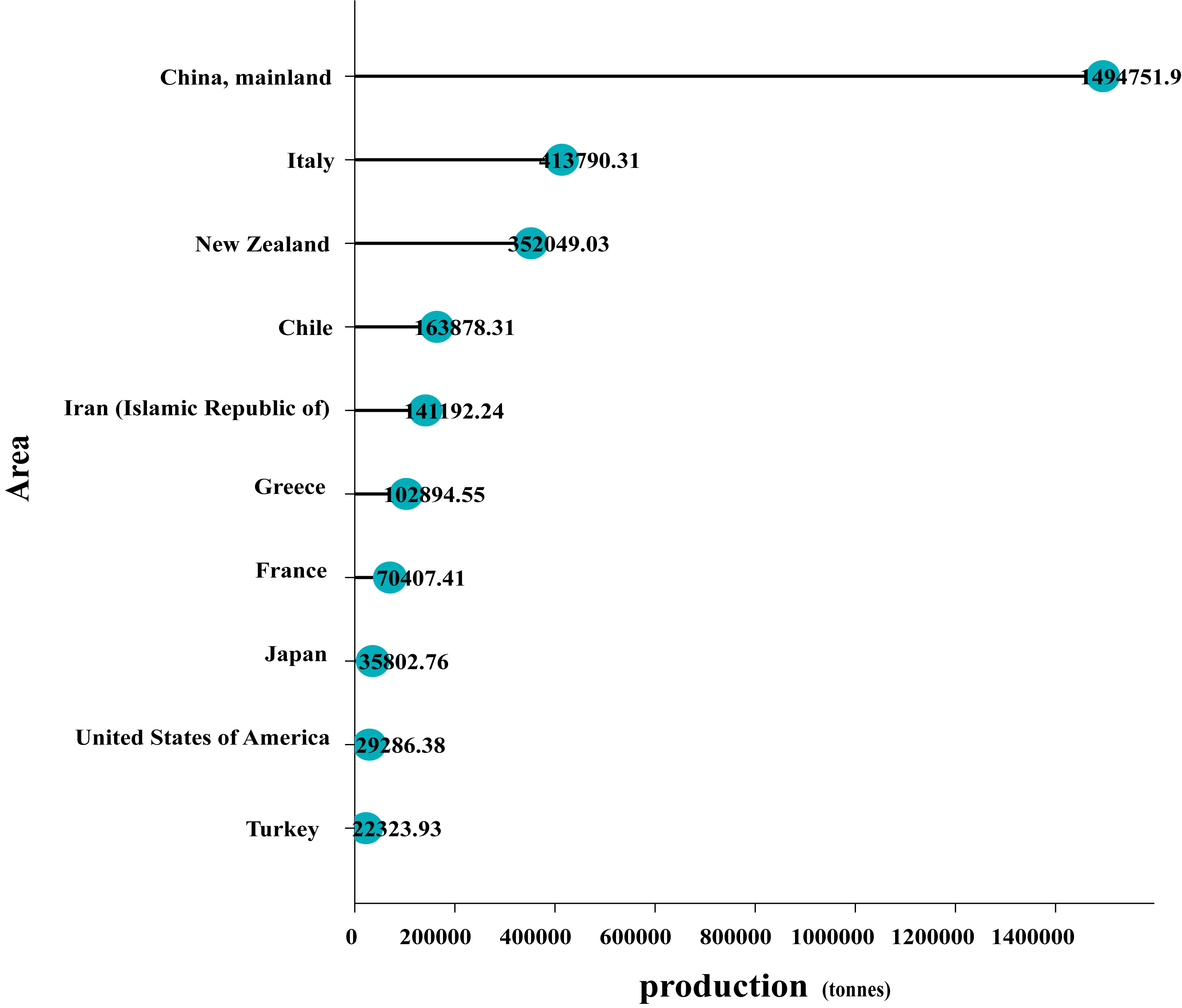

Supplement: Supplementary file 1 [file genes-13-01827-s001.zip › Figure S5 Production of kiwifruit top 10 producers.tif]
